# Intervention to Match Young Black Men and Transwomen Who Have Sex With Men or Transwomen to HIV Testing Options (All About Me): Protocol for a Randomized Controlled Trial

**DOI:** 10.2196/resprot.8856

**Published:** 2017-12-19

**Authors:** Beryl Koblin, Sabina Hirshfield, Mary Ann Chiasson, Leo Wilton, DaShawn Usher, Vijay Nandi, Donald R Hoover, Victoria Frye

**Affiliations:** ^1^ Laboratory of Infectious Disease Prevention New York Blood Center New York, NY United States; ^2^ Public Health Solutions New York, NY United States; ^3^ Department of Human Development Binghamton University Binghamton, NY United States; ^4^ Faculty of Humanities University of Johannesburg Johannesburg South Africa; ^5^ Laboratory of Data Analytic Services New York Blood Center New York, NY United States; ^6^ Department of Statistics and Biostatistics Institute for Health, Health Care Policy and Aging Research Rutgers University Piscataway, NJ United States; ^7^ Community Health and Social Medicine Department Sophie Davis School of Biomedical Education/CUNY School of Medicine City College of New York, City University of New York New York, NY United States

**Keywords:** HIV testing, black men who have sex with men, transgender women, mobile technology, HIV prevention

## Abstract

**Background:**

HIV testing is a critical component of HIV prevention and care. Interventions to increase HIV testing rates among young black men who have sex with men (MSM) and black transgender women (transwomen) are needed. Personalized recommendations for an individual’s optimal HIV testing approach may increase testing.

**Objective:**

This randomized trial tests the hypothesis that a personalized recommendation of an optimal HIV testing approach will increase HIV testing more than standard HIV testing information.

**Methods:**

A randomized trial among 236 young black men and transwomen who have sex with men or transwomen is being conducted. Participants complete a computerized baseline assessment and are randomized to electronically receive a personalized HIV testing recommendation or standard HIV testing information. Follow-up surveys are conducted online at 3 and 6 months after baseline.

**Results:**

The All About Me randomized trial was launched in June 2016. Enrollment is completed and 3-month retention is 92.4% (218/236) and has exceeded study target goals.

**Conclusions:**

The All About Me intervention is an innovative approach to increase HIV testing by providing a personalized recommendation of a person’s optimal HIV testing approach. If successful, optimizing this intervention for mobile devices will widen access to large numbers of individuals.

**Trial Registration:**

ClinicalTrial.gov NCT02834572; https://clinicaltrials.gov/ct2/show/NCT02834572 (Archived by WebCite at http://www.webcitation.org/6vLJWOS1B)

## Introduction

Men who have sex with men (MSM) comprised the largest proportion (67%) of new HIV diagnoses in the United States in 2015 [1]. Young black MSM are diagnosed with HIV at greatly disproportionate rates, comprising 50% of all new diagnoses among MSM aged 15 to 29 years [1]. The prevalence and incidence of HIV have repeatedly been demonstrated to be high among young black MSM [2-4]. Furthermore, high HIV prevalence and incidence rates have been reported among transgender women (transwomen), with black transwomen disproportionately affected [5,6].

A key component of HIV prevention for communities at risk is regular HIV testing. Persons unaware that they are HIV-infected cannot benefit from antiretroviral treatment and are more likely to transmit HIV to others due to lack of viral suppression [7]. Testing also provides gateways to HIV prevention strategies, such as pre-exposure prophylaxis (PrEP). The Centers for Disease Control and Prevention (CDC) recommends that MSM test for HIV at least annually and more frequently (every 3-6 months) if they have additional risk factors [8,9]. Although reports indicate that HIV testing has increased among young black MSM (eg, 65% testing in last 12 months in 2008 to 77% in 2011) [10], the need to further increase HIV testing, uptake of prevention strategies and linkage to care early in HIV infection continues for young black MSM and transwomen [5,11,12]. For example, in a cohort of black MSM and transwomen, only 28% of those queried in 2015 were aware of PrEP [13] and the CDC reports that young black MSM have significantly less favorable HIV care outcomes than do others; 66% of black MSM aged 20-24 years are linked to care within 1 month of diagnosis compared to 77% of white MSM in the same age group [14].

General strategies to increase HIV testing include community-based campaigns, opt-out policies at clinics, electronic medical record alerts, and offering testing in various outreach venues [15-19]. A limited number of HIV testing interventions among young black MSM and transwomen have been reported. One intervention that focused specifically on HIV testing among young black MSM found that a field outreach approach combined with motivational interviewing was associated with a higher uptake of HIV testing and returning for test results [20]. Of several peer-led interventions, a group-level, culturally congruent, theory-based behavior change intervention for older black MSM resulted in a significant increase in self-reported HIV testing [21].

Mobile technology and Web technology, as well as text messaging HIV prevention interventions, have been developed for young MSM, with some studies focused on HIV testing uptake as an outcome [22-28]. Distribution of self-test kits through social media promotions has also been examined as an approach to increase HIV testing [29,30]. Merchant et al [31] found that access to free self-tests resulted in the highest uptake of HIV testing, even among those for whom this was not their preferred testing approach.

To our knowledge, no interventions designed to increase HIV testing are oriented toward taking advantage of the variety of HIV testing approaches now available, including traditional clinic/doctor/community-based testing, self-testing [32], and couples HIV testing and counseling [33]. Multiple testing approaches provide the opportunity for a personalized intervention, which can enable user choice and increase levels of uptake as demonstrated with multiple technologies in the contraceptive field [34].

This randomized controlled trial was developed with the goal of increasing HIV testing among young black MSM and transwomen by providing a personalized recommendation for an individual’s optimal HIV testing approach. The intervention is a brief survey completed by participants, which provides the data for an algorithm to make the personalized recommendation. The intervention integrates available HIV testing options, including HIV self-tests for those who may be unable or unwilling to visit a testing site; couples HIV testing and counseling, given data suggesting that a significant proportion of HIV transmission among MSM may be attributed to sex with main partners [35,36]; and traditional clinic-based testing for those most comfortable testing in such a setting. The intervention also takes advantage of the widespread use of the Internet and mobile devices by young black MSM and transwomen, including those who are otherwise hard to reach [37]. This approach has potential to reach large numbers of young black MSM and transwomen, especially those who are unlikely to attend public venues, are unable or unwilling to come to traditional testing sites, and are at risk but are less likely to have recently tested for HIV.

## Methods

### Design

A trial is being conducted among 236 young black MSM and transwomen randomized to receive either a personalized recommendation electronically (eg, on their computer or mobile screen) for an HIV testing approach (intervention) or standard HIV testing information (control). At 3 and 6 months, standardized online surveys assess outcomes and covariates. This randomized trial tests the hypothesis that the personalized recommendation increases HIV testing more than standard testing information.

### Participants

The original study design limited enrollment to black MSM aged 18 to 29 years. Based on feedback from the study Community Consulting Group and their knowledge of HIV risk among black subpopulations in New York City, inclusion criteria were expanded to include those aged 16 and 17 years and transwomen. Thus, participants are eligible if they (1) are a male at birth; (2) self-identify as black, African American, Caribbean black, African black, or multiethnic black; (3) are able to read and respond in English; (4) are aged between 16 and 29 years; (5) are not known to be HIV-infected; (6) report insertive or receptive anal intercourse with a man or transwoman in the last 12 months; (7) reside within the New York City metropolitan area; (8) are willing to participate in a study for 6 months; (9) have a working email address and phone number to receive texts, calls, or emails for follow-up data collection; and (10) provide informed consent or assent for the study. Participants are ineligible if they are enrolled in any other HIV-related research study involving HIV testing, have been a participant in an HIV vaccine trial, or are currently taking PrEP.

Recruitment occurs via online advertising, face-to-face outreach, and referrals by study participants. For online recruitment, persons who click on an ad and are deemed eligible from a brief eligibility assessment on the study website are asked to complete a short online contact card. For face-to-face recruitment, potentially eligible individuals are told about the study and given a brief eligibility assessment. Those who are deemed eligible are asked to provide a phone number or email address for study staff follow-up. If screening for eligibility is not possible, staff collect contact information so the person can be screened over the telephone. Participants can refer up to three people for the study and receive US $10 for each person who attends a baseline visit. Staff use the contact information from interested and preliminarily eligible persons to email, text, or call to schedule a study visit.

### Study Procedures

**Figure 1:**
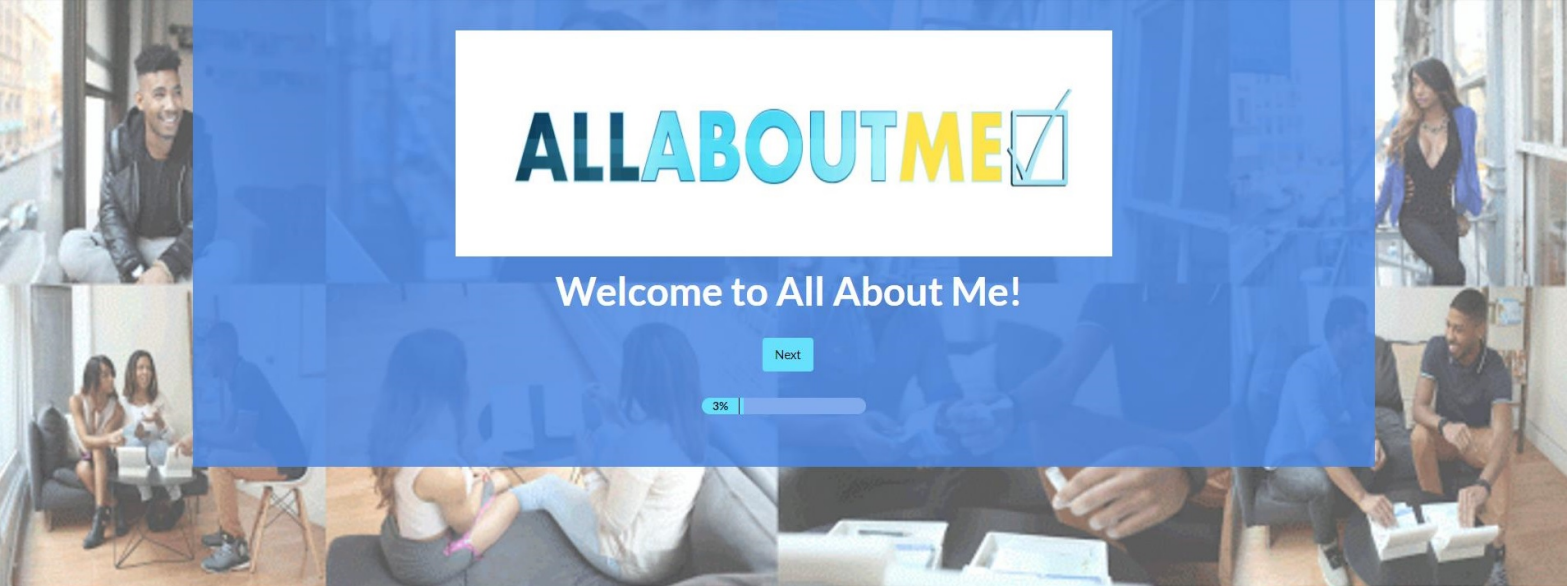
All About Me platform introductory page (persons shown in photographs are models and are not actual study participants).

**Figure 2:**
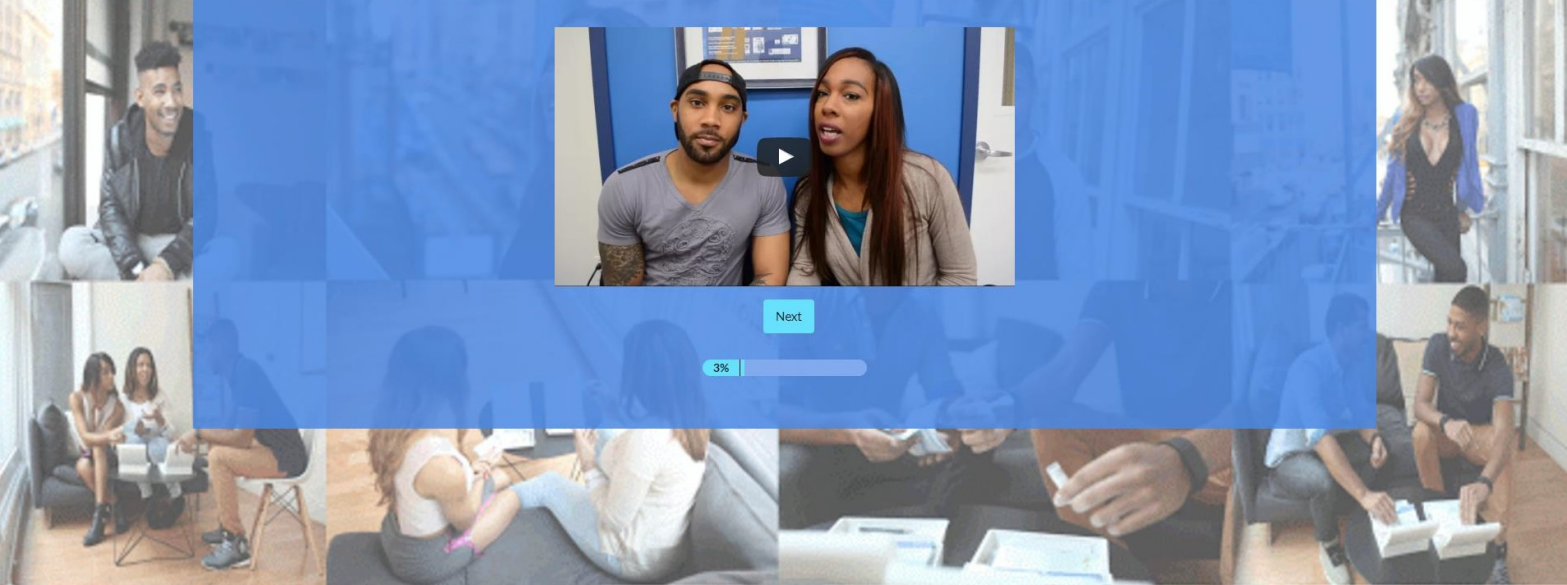
Still from video describing All About Me trial.

After giving informed consent, participants are asked to provide extensive contact information to assist with study retention. Participants are then introduced to the All About Me computer-based platform with short videos describing the study (Figures 1 and 2; Multimedia Appendix 1: What is All About Me?).

**Figure 3:**
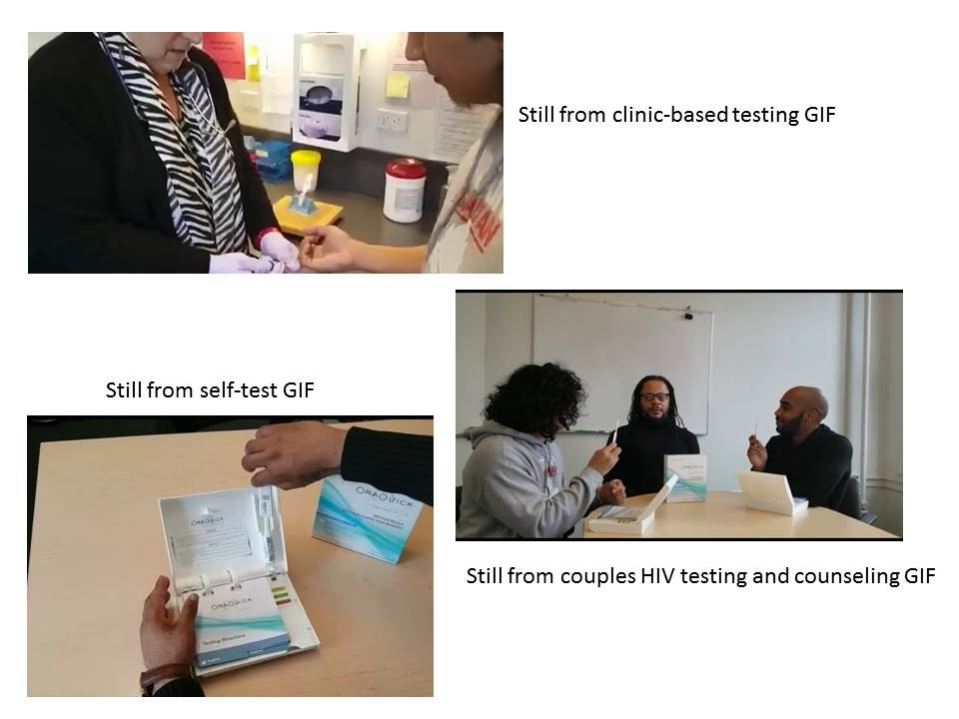
Stills from GIFs describing HIV testing approaches.

The platform is comprised of four sections: (1) staff-only section for entering participant ID number and randomization assignment; (2) a quantitative baseline assessment; (3) information about each HIV testing method (each represented with a GIF; Figure 3), including how the tests are conducted (blood, oral sample) and availability of counseling and support; and (4) the intervention or control conditions (described subsequently).

The baseline assessment covers demographics; HIV testing history, including use of self-testing and couples HIV testing and counseling; and sexual risk behaviors and substance use in the prior 3 months. Questions about sexual behaviors in the prior 3 months include the number of anal or vaginal sex partners, insertive and receptive anal sex, condom use, and HIV status of partners. Questions about use of substances in the prior 3 months include marijuana, stimulants (powder cocaine, crack cocaine, methamphetamine), prescription drugs, poppers, erectile dysfunction drugs, and club drugs [38]. Presence of depressive symptoms is measured with the Patient Health Questionnaire 2 (PHQ-2) [39]. HIV stigma is measured with a scale of eight items, such as “If you talk too much about HIV, people will think that you have HIV” with a four-point response scale of strongly disagree to strongly agree (α=.88) [40]. Participants receive US $25 cash for completion of the baseline visit.

### Randomization

Participants are randomized in a 1:1 ratio into one of the two study arms. Randomly ordered block sizes of four and six stratified by age (16-23 and 24-29 years) are generated by the study data analyst (VN) using Sealed Envelope Ltd [41]. Assignments are placed in sequentially numbered, opaque, sealed envelopes. After informed consent, the next envelope is opened and the staff member enters the participant’s study ID number and the assignment into the All About Me study platform. Neither staff nor participants are blinded to study arm assignment.

### Follow-Up Surveys

For assessment of primary and secondary outcomes as well as covariates, participants are sent a link to 3- and 6-month follow-up surveys by email. The quantitative assessment includes the same questions as in the baseline survey, excluding some demographics. After the final survey at 6 months, all participants are offered the intervention algorithm to provide them with a personalized HIV testing approach. If a participant reports testing HIV positive in one of the follow-up surveys, the participant remains in the study and staff contacts the participant to provide resources for linkage to care and social services, as needed.

### Incentives

To provide the participants with a choice of gift cards from a range of companies, we contract with a promotion code redemption site to distribute a US $25 e-gift card for completion of the 3-month survey and a US $30 e-gift card for completion of the 6-month survey.

### Intervention Development

The intervention is modeled after a successful computer-based assessment to improve contraceptive method choice and continuation of use among women at urban publicly funded family planning centers [42,43]. To inform the development of the intervention, a formative mixed method research phase of in-depth interviews and quantitative surveys was conducted, framed broadly within social cognitive theory [44,45] and several midrange theories, including the theory of planned behavior [46], stigma theory [47], social identity theory [48], and social norms theory [45]. In-depth interviews identified barriers and facilitators to HIV testing and knowledge about HIV testing approaches (self-testing, couples HIV testing and counseling, or clinic-based testing) among young black MSM and transwomen with a particular focus on the potential influence of sociodemographic, psychosocial, mental health, and sociostructural factors on HIV testing uptake, and assessed participant perspectives on the intervention that may enhance its effectiveness in increasing HIV testing [49].

The computerized algorithm that provides a personalized recommendation of the optimal HIV testing approach for individuals was based on the findings from a quantitative survey described previously [38]. The survey collected data on intentions to test by self-test, couples HIV testing and counseling, and at a clinic or other provider, as well as awareness and comfort levels with specific testing modalities, sociostructural factors, behavioral risk, peer norms, social support, and stigma [38]. Stepwise selection multivariable modeling identified variables statistically independently associated with intention to test by each of three specific HIV testing methods (Table 1; reported previously in [38]). In the intervention section of the All About Me platform, three probabilities of intention to test from this algorithm are calculated for each participant, one for each testing method. We developed decision rules that use these probabilities for calculating that participant’s personalized HIV testing approach. Specifically, the testing approach with the highest probability for an individual is the recommended testing approach. If the probability for all three testing approaches is less than .45 (suggesting that none of the methods are strongly indicated) then testing at a clinic or other provider is recommended. If the probabilities for two approaches are within .05 of each other, then the recommendation is to test by either method.

Forty-two case studies with varied combinations of factors associated with testing were presented to the research team members who were not involved in the quantitative survey analysis to compare their recommendation to the computer algorithm. For example, one case was a young black MSM with a high school education and who has health insurance. He is comfortable testing at home with a friend or partner. He has a low level of social support and HIV testing self-efficacy. He cites stigma/fear as a reason not to test. Using the algorithm and the decision rules, this individual would receive a recommendation of self-testing. There were no cases in which the algorithm recommendation was determined to be an unacceptable recommendation by the team, even when taking into account case study psychosocial (eg, social support) and structural issues (eg, incarceration history).

Once the initial intervention interface was developed, feedback was obtained from the study Community Consulting Group and two focus groups of young black MSM and transwomen. A second Community Consulting Group meeting and two additional focus groups were held to demonstrate the intervention and to obtain feedback on the graphics and usability of the online intervention. For the final step in the intervention development, a small pilot study was conducted with 10 young black MSM and transwomen to obtain data on the usability of the online intervention. The participants tested the intervention at the study site and had a debriefing interview with trained study staff to describe the acceptability, clarity, and utility of intervention (eg, experience with the study platform, attractiveness of the graphics and layout, culturally appropriate language, clear HIV testing information, ease of use). Final changes to the intervention were completed based on pilot results.

**Table 1:** Stepwise selection multivariable analysis for intention to test by specific testing approaches, All About Me Study (N=169).

Variables	Intention to test by..., aOR (95% CI)^a^		
	Self-test^b^	Clinic or other provider^c^	Couples HIV testing and counseling^b^
Comfort in testing by a friend or partner at home	2.4 (1.1-5.3)		
Stigma or fear as a reason not to test	8.6 (2.5-29.7)		
Social support (per point higher)	0.5 (0.3-0.7)	2.0 (1.3-2.9)	
Health insurance	0.2 (0.1-0.5)		
Self-efficacy for HIV testing (per point higher)		2.9 (1.5-5.6)	
Lifetime history of incarceration		0.4 (0.2-0.9)	
Some college/associate’s degree vs high school grad or less			0.8 (0.4-1.7)
Bachelor’s degree or higher vs high school grad or less			0.3 (0.1-0.7)
Have a primary partner			1.8 (1.0-3.5)

^a^aOR: adjusted odds ratio.

^b^Self-test and couples HIV testing and counseling outcomes: very likely / somewhat likely versus somewhat unlikely / very unlikely.

^c^Clinic outcome: very likely versus somewhat likely / somewhat unlikely / very unlikely.

**Figure 4:**
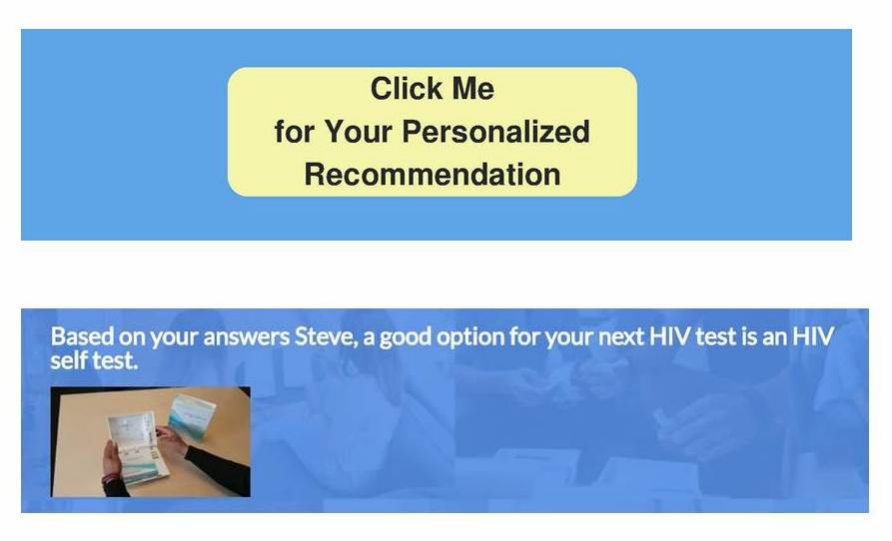
Personalized HIV testing approach.

### Intervention and Control Conditions

#### Intervention Arm

Participants assigned to the intervention arm answer questions to assess factors required to complete the algorithm (Table 1: educational level, health insurance, incarceration history, primary partner, stigma or fear as a reason not to test, HIV testing self-efficacy scale, comfort testing with a friend or partner at home, social support scale). They then receive the results of the HIV testing algorithm, presented as their personalized HIV testing approach (Figure 4).

Participants recommended to clinic-based testing are presented with a widget [50] to find an HIV testing site. The widget can be sent to their phone via email or text. Participants recommended to the self-testing approach are given one of three options to receive a free self-test kit: (1) buy the kit and bring the receipt to the study site for reimbursement, (2) receive a code by text or email to order a test kit online to be mailed to them from OraSure, or (3) receive a coupon by text or email to pick up a self-test kit at the study site. Participants recommended to couples HIV testing and counseling are given a listing of free HIV testing sites that offer couples HIV testing and counseling. The list can also be sent to their phone by email.

#### Control Arm

Control participants are provided with information about ways to test by each testing approach but without a recommended approach. Thus, they are presented with all the following: a widget to find an HIV testing site that can be sent to their phone via email or text, information on how to obtain a self-test kit, and a list of free HIV testing sites that offer couples HIV testing and counseling that can also be sent to their phone by email.

To reduce the potential bias of the cost of self-test kits as a factor in future HIV testing, when an intervention participant is recommended a HIV self-test kit and offered a free kit, the next control participant in the same age strata is also offered a free self-test kit.

### Outcomes

The primary outcome is self-reported occurrence of HIV testing during 6 months of follow-up. Secondary outcomes include use of HIV self-testing and couples HIV testing and counseling and, for intervention participants, testing methods used compared to algorithm-recommended methods.

### Statistical Analysis

Analysis of outcomes will be conducted on an intention-to-treat basis. Descriptive analyses will identify outliers (including potential erroneous values), need for variable transformations to enable statistical comparison, and baseline comparability of treatment arms. To account for potential biases resulting from those who discontinue participation, dropouts will be compared to completers by intervention and control group assignment with respect to baseline behavior and other characteristics to test whether differential dropout could influence results. Sensitivity analyses in which dropouts are considered failures will also be done.

Contingency tables (with odds ratios, relative risks, and exact tests) or means/medians (with *t* tests or rank tests) will be used to compare baseline characteristics among study arms. For each time point considered, the binary outcome of primary interest will be compared between the intervention and control arms using contingency tables with odds ratios, relative risks, and exact tests. Pooled analyses of multiple time points will be done using generalized estimation equations with logit link and the person as a cluster. If time to event is indicated, then Kaplan-Meir, proportional hazards, or discrete logistic regression analogs will be used. Comparisons will be adjusted for other covariates as needed using linear/logistic regression or proportional hazards models, as appropriate. Analyses will be stratified by specific covariates to test for modifying effects on the intervention, when needed. Similar approaches will be used to compare dropout and missing longitudinal data between intervention arms, and if these differ between study arms, a sensitivity analysis approach will be used to adjust for potential impact of this on comparisons of the study outcomes.

### Sample Size

Hypothesis testing is two-sided with α=.05. We have enrolled 236 participants (118 per arm) and we estimate 15% attrition at 6 months and thus 100 participants per arm. With a range of testing uptake of 20% to 50% in the control arm, we have 80% power to detect an increase of at least 23% (ie, from 20% to 43% or 50% to 83%) in HIV testing uptake in the intervention arm [51].

### Data Safety Monitoring Board

A Data Safety Monitoring Board (DSMB) has been convened, comprised of experts in HIV testing related to our priority population of black MSM and transwomen. All members are independent of the trial and funding agency. The DSMB determines safe and effective conduct of the trial and recommends conclusion of the trial if significant risks develop or if the trial is unlikely to finish successfully. Monitoring calls occur every 6 months and evaluation is conducted of the progress of the trial, including periodic assessments of participant recruitment, accrual and retention, participant risk versus benefit, and other factors that may affect study outcomes. Monitoring may also consider factors external to the study when interpreting the data, such as scientific developments that may have an impact on the safety of the participants or ethical issues related to the study.

### Institutional Review Board Approval

The study was approved and is reviewed annually by the Institutional Review Boards of the New York Blood Center, Public Health Solutions, and Binghamton University. A Certificate of Confidentiality was obtained from the funder.

## Results

**Figure 5:**
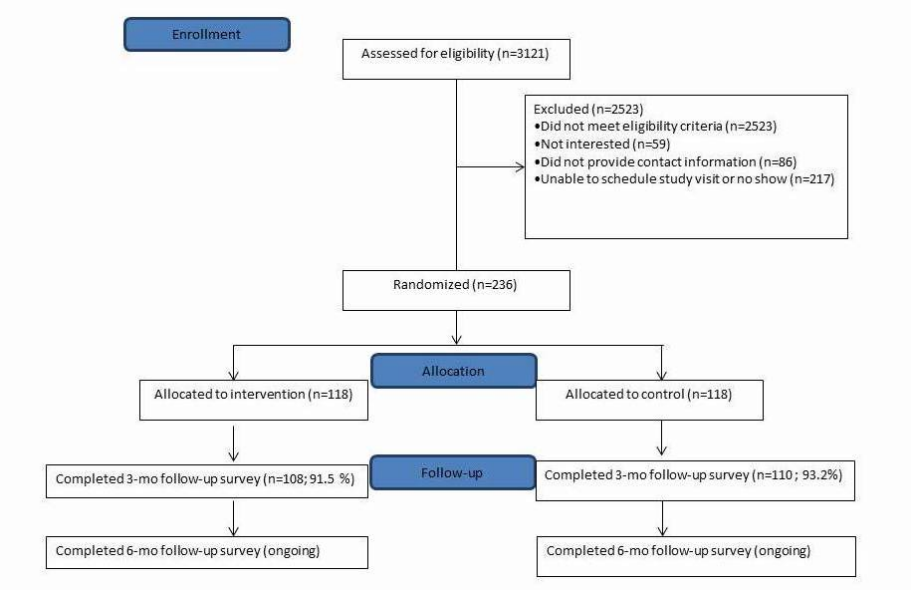
CONSORT flow diagram.

The trial was initiated in June 2016. Enrollment and randomization of 236 participants were completed in February 2017 with 118 participants randomized to the intervention arm and 118 to the control arm (Figure 5). Retention for follow-up surveys has been high. The visit windows for the 3-month surveys are now closed, with a retention rate of 92.4% (218/236). Six-month surveys are ongoing and the current retention among those with closed visit windows is 93.0% (107/115).

## Discussion

The HIV epidemic among young black MSM and transwomen in the United States is an urgent public health problem. HIV diagnosis is essential for care and treatment and to reduce HIV transmission; regular HIV testing is critical for HIV prevention among persons at risk.

To this end, an option such as self-testing may increase HIV testing among young black MSM and transwomen by allowing them to test privately, at their convenience, alleviating the burden of having to access traditional testing venues, which carry potential stigma and confidentiality concerns. At the same time, for some, self-testing may not be appropriate (eg, lack of privacy at home, need for social support), thus warranting testing at an HIV testing site with a counselor. Alternatively, couples HIV testing and counseling by a trained counselor could provide the opportunity to test and receive test results with a partner, a potential source of social support.

In this study design, there are some limitations. We considered a longer follow-up period to address the issue of sustainability, but a mobile device intervention will likely have more immediate effects. The primary outcome is self-reported. It would have been difficult to verify HIV testing given the wide range of testing options and venues available.

The All About Me intervention provides an innovative approach that ties these options together and gives individuals a personalized recommendation of their likely optimal HIV testing approach, supported by evidence from the contraceptive field that personalized recommendations increase uptake, coverage, and adherence [42,43]. To access HIV prevention and care, increasing HIV testing as a gateway behavior is crucial. The All About Me intervention optimized for mobile devices and integrated into HIV prevention and care programs provides an opportunity to access large numbers of individuals, especially those who may be less likely to have recently tested and may not otherwise be reached.

## Appendix

Multimedia Appendix 1 What is All About Me?

## Abbreviations

CDC: Centers for Disease Control and Prevention
DSMB: Data Safety Monitoring Board
MSM: men who have sex with men
PHQ-2: Patient Health Questionnaire 2
PrEP: pre-exposure prophylaxis
